# Real-world outcomes of stereotactic body radiotherapy plus sintilimab and bevacizumab for hepatocellular carcinoma with portal vein tumor thrombus

**DOI:** 10.1093/oncolo/oyaf439

**Published:** 2026-01-06

**Authors:** Bin Zhou, Zihui Ma, Lei Wang, Yue Lu, Xi Cheng, Yabo Jiang, Xubiao Wei, Chongde Lu, Shuqun Cheng, Xingsheng Lu

**Affiliations:** Department of Hepatopancreatobiliary Surgery, The Fourth Affiliated Hospital of Soochow University, Suzhou, 215000, China; Department of Hepatic Surgery VI, Eastern Hepatobiliary Surgery Hospital, Naval Medical University, Shanghai, 200438, China; Department of Hepatic Surgery VI, Eastern Hepatobiliary Surgery Hospital, Naval Medical University, Shanghai, 200438, China; Department of Hepatic Surgery VI, Eastern Hepatobiliary Surgery Hospital, Naval Medical University, Shanghai, 200438, China; Department of Hepatopancreatobiliary Surgery, The Fourth Affiliated Hospital of Soochow University, Suzhou, 215000, China; Department of Radiotherapy, Eastern Hepatobiliary Surgery Hospital, Naval Medical University, Shanghai, 200438, China; Department of Hepatic Surgery VI, Eastern Hepatobiliary Surgery Hospital, Naval Medical University, Shanghai, 200438, China; Department of Hepatic Surgery VI, Eastern Hepatobiliary Surgery Hospital, Naval Medical University, Shanghai, 200438, China; Department of Hepatic Surgery VI, Eastern Hepatobiliary Surgery Hospital, Naval Medical University, Shanghai, 200438, China; Department of Hepatic Surgery VI, Eastern Hepatobiliary Surgery Hospital, Naval Medical University, Shanghai, 200438, China; Department of Hepatopancreatobiliary Surgery, The Fourth Affiliated Hospital of Soochow University, Suzhou, 215000, China

**Keywords:** hepatocellular carcinoma, portal vein tumor thrombus, sintilimab, bevacizumab, stereotactic body radiotherapy, real-world study

## Abstract

**Background:**

Sintilimab plus bevacizumab (Sin + Bev) is recommended as a first-line treatment for hepatocellular carcinoma (HCC) with portal vein tumor thrombus (PVTT); however, its efficacy remains limited. This study aimed to assess the efficacy and safety of stereotactic body radiotherapy (SBRT) combined with Sin + Bev for HCC with PVTT.

**Patients and Methods:**

This retrospective study included 69 HCC patients with PVTT treated at two centers in China from August 2021 to December 2022. Patients received either SBRT + Sin + Bev (*n* = 31) or Sin + Bev alone (*n* = 38). Primary endpoints were overall survival (OS) and progression-free survival (PFS). Secondary endpoints included objective response rate (ORR) and treatment-related adverse events (TRAEs).

**Results:**

Baseline characteristics showed similar distributions between the two groups. SBRT + Sin + Bev group showed a significant increase in both median OS (18.9 vs 9.3 months, *P* < .001), median PFS (9.1 vs 4.7 months, *P* = .0015), and ORR (64.6% vs 26.3%, *P* = .031) compared to Sin + Bev group. TRAEs in SBRT + Sin + Bev group were mostly manageable and acceptable compared to Sin + Bev group. Multivariate analysis confirmed that SBRT + Sin + Bev was an independent prognostic factor for better OS (*P* < .001) and better PFS (*P* < .001).

**Conclusion:**

SBRT combined with sintilimab plus bevacizumab appears to improve treatment efficacy and demonstrates a favorable safety and feasibility profile for HCC with PVTT.

Implications for Practice The presence of portal vein tumor thrombus (PVTT) in hepatocellular carcinoma (HCC) is a key factor contributing to poor overall prognosis. Systemic therapy alone offers limited benefit for those patients. This study demonstrates that stereotactic body radiotherapy (SBRT) combined with sintilimab and bevacizumab significantly improves survival in HCC patients with PVTT, without increasing treatment-related toxicity. These findings suggest that this triple modality approach could offer a therapeutic option for HCC with PVTT.

## Introduction

Hepatocellular carcinoma (HCC) is a common malignant tumor that is highly lethal and poses a significant global health burden.^1^ The presence of portal vein tumor thrombus (PVTT) in HCC is a key factor contributing to poor prognosis, with a median survival of less than 4 months without treatment.^2^^,^^3^ Systemic therapy is recommended for HCC with PVTT, with immunotherapy plus anti-angiogenic agents now considered a novel first-line option.^4-6^ Sintilimab plus bevacizumab (Sin + Bev) is the first-line treatment for HCC and is widely used across China.^3^^,^^5^ However, the prognosis with systemic therapy alone remains unsatisfactory, especially for patients with PVTT, highlighting the urgent need to explore more effective treatment strategies.

Radiotherapy (RT) is an effective treatment approach for HCC with PVTT, as PVTT is sensitive to RT.^7^ Besides, stereotactic body radiotherapy (SBRT) delivers high-dose radiation precisely to tumors while sparing nearby healthy tissue. Therefore, SBRT has demonstrated improved local control rates in treating PVTT compared to other RT methods.^8^ Besides inflicting tumor cell apoptosis, RT also enhances the infiltration of immune cells.^9^ Furthermore, RT can augment the effectiveness of the targeted drug by stimulating the expression of anti-angiogenic targets within the microenvironment.^10^ Hence, the addition of RT to systemic therapy may yield a synergistic effect.

Recent retrospective studies have indicated that a triple combination therapy involving immunotherapy, targeted agents, and intensity modulated radiation therapy (IMRT) can yield positive results in advanced HCC.^11-13^ A recent clinical trial found that combining IMRT with Sin + Bev could improve the prognosis of HCC patients.^14^ However, to date, no published studies have reported the combination of SBRT with Sin + Bev as first-line treatment for HCC with PVTT. Hence, this retrospective real-world study was conducted to preliminarily assess the feasibility and safety of this therapeutic strategy.

## Methods

### Patient enrollment

Clinical data were retrospectively collected from two centers (The Fourth Affiliated Hospital of Soochow University and Eastern Hepatobiliary Surgery Hospital) between August 2021 and December 2022. The inclusion criteria were: (1) clinically or histologically diagnosed HCC with PVTT, (2) receive Sin + Bev treatment with or with SBRT, (3) Child-Pugh A or B, (4) ECOG PS 0-1, (5) at least one measurable liver lesion ≥1 cm or PVTT >1 cm. The exclusion criteria were: (1) combination with TACE or radiofrequency ablation, (2) previous receipt of any systemic treatment, (3) presence of extrahepatic metastasis, (4) absence of baseline examination, and (5) loss to patient follow-up. The research was approved by the Institutional Ethics Committee (No. 2024-241135).

According to Cheng’s PVTT classification, PVTT is classified into four types: type I, tumor thrombus involving at least the secondary portal vein; type II, the right portal vein or left portal vein; type III, the main portal vein; type IV, the superior mesenteric vein.^3^

### Treatment protocol

Radiation oncologists utilize four-dimensional computed tomography (CT) simulation to determine treatment positioning, outlining the contour of the gross tumor volume (GTV) and identifying organs at risk (OARs). GTV typically encompasses the total volume of HCC and PVTT. If the patient’s tumor is large or has multiple intrahepatic metastases, GTV refers to PVTT. Expand the GTV by 3-5 mm to obtain the planning target volume (PTV) and ensure that it does not overlap with adjacent OARs. The dose for the PTV was calculated with the CyberKnife Multiplan system (v4.0.2). The AAPM TG-101 report^15^ provided guidance on the tolerance doses for the OARs. There were five patients who received SBRT targeting HCC and PVTT (40 Gy/5 fx), while 26 patients received SBRT targeting PVTT (36 Gy/4-5 fx).

For the SBRT + Sin + Bev group, patients initiate Sin + Bev treatment 3 ± 1 day after completing the final SBRT session. For the Sin + Bev group, sintilimab (200 mg) is administered intravenously slowly (over 60 min), followed by intravenous infusion (over 90 min) of bevacizumab (15 mg/kg). Patients received Sin + Bev every 3 weeks as a single cycle. According to the manufacturer’s instructions, treatment may be adjusted or interrupted if patients experience ≥Grade 3 adverse reactions or cannot tolerate drug-related side effects until the drug-related adverse reactions decrease to Grade 1 or disappear.

### Response and safety evaluation

Based on the initial images, the overall tumor response is assessed utilizing the Modified Response Evaluation Criteria in Solid Tumors (mRECIST) criteria.^16^ Target lesions were defined as intrahepatic tumor nodules ≥1 cm in the longest diameter that showed intratumoral arterial enhancement. Up to two such lesions were selected as target lesions for each patient. PVTT was considered a nontarget lesion. Objective response rate (ORR) = partial response (PR) + complete response (CR), while disease control rate (DCR) = stable disease (SD) + ORR. CR required both HCC and PVTT to achieve CR; PD was defined as progression in HCC and/or PVTT; SD was defined as HCC with stable disease and PVTT non-PD; all other responses were classified as PR. Assessments were performed every 6 weeks. Treatment-related adverse events (TRAEs) are assessed using the Common Terminology Criteria for Adverse Events (CTCAE) (version 5.0).^17^

### Follow-up

After initiation of Sin + Bev treatment, follow-up assessments were conducted every 2 to 3 months thereafter until either patient death or the data cutoff date (December 31, 2024), whichever occurred first. Each follow-up included evaluation of the patient’s general condition, blood tests (complete blood count, liver function, AFP, coagulation profile, etc), and radiological evaluation (chest CT and liver CT or MRI). Overall survival (OS) refers to the period from the start of Sin + Bev treatment to either death from any cause or the latest follow-up. Progression-free survival (PFS) refers to the period from the start of Sin + Bev treatment to death from any cause or the first occurrence of radiologically confirmed disease progression, according to mRECIST criteria.

### Statistical analysis

Comparison of numeric variables across groups was conducted through independent *t*-tests, while categorical variables were assessed using chi-square or Fisher’s exact tests. Survival rates were estimated using the Kaplan–Meier method. Survival outcomes were compared through univariate and multivariate Cox regression analysis. Variables (*P*-value <.10) were included in the additional analysis to calculate hazard ratios (HR) and 95% confidence intervals (95% CI) for determining statistical significance. Statistical analyses were carried out using R (v4.3.1) and SPSS (v27.0). A *P*-value <.05 was considered statistically significant.

## Results

### Patient characteristics

As shown in Figure S1, 69 patients were included in the final analysis after screening. There were 31 patients in the SBRT + Sin + Bev group and 38 patients in the Sin + Bev group. Baseline characteristics showed similar distributions between the SBRT + Sin + Bev group and the Sin + Bev group (Table 1). The majority of patients in the cohort (91.3%, 63/69) were infected with the hepatitis B virus (HBV).

**Table 1.** Baseline characteristics of the study population.

Characteristics	**Total enrolled (*n*** =** 69)**	Sin + Bev (*n* = 38)	SBRT + Sin + Bev (*n* = 31)	*P*
**Gender, *n* (%)**				
** Male**	63 (91.3)	37 (97.4)	26 (83.9)	.121
** Female**	6 (8.7)	1 (2.6)	5 (16.1)	
**Age, *n* (%)**				
** ≥55 years**	39 (56.5)	19 (50.0)	20 (64.5)	.226
** <55 years**	30 (43.5)	19 (50.0)	11 (35.5)	
**HBV, *n* (%)**				
** Yes**	63 (91.3)	36 (94.7)	27 (87.1)	.490
** No**	6 (8.7)	2 (5.3)	4 (12.9)	
**Child–Pugh, *n* (%)**				
** A**	57 (82.6)	31 (81.6)	26 (83.9)	.803
** B**	12 (17.4)	7 (18.4)	5 (16.1)	
**ECOG, *n* (%)**				
** 0**	35 (50.7)	21 (55.3)	14 (45.2)	.404
** 1**	34 (49.3)	17 (44.7)	17 (54.8)	
**Tumor number, *n* (%)**				
** =1**	35 (50.7)	16 (42.1)	19 (61.3)	.113
** >1**	34 (49.3)	22 (57.9)	12 (38.7)	
**Tumor size, *n* (%)**				
** <5 cm**	13 (18.8)	7 (18.4)	6 (19.4)	.298
** 5-10 cm**	34 (49.3)	16 (42.1)	18 (58.1)	
** >10 cm**	22 (31.9)	15 (39.5)	7 (22.6)	
**AFP, *n* (%)**				
** <200 ng/mL**	37 (53.6)	17 (44.7)	20 (64.5)	.101
** ≥200 ng/mL**	32 (46.4)	21 (55.3)	11 (35.5)	
**WBC, *n* (%)**				
** <4 × 10^9^/L**	30 (43.5)	16 (42.1)	14 (45.2)	.799
** ≥4 × 109/L**	39 (56.5)	22 (57.9)	17 (54.8)	
**ALBI, *n* (%)**				
** 1**	10 (14.5)	6 (15.8)	4 (12.9)	1.000
** 2**	59 (85.5)	32(84.2)	27 (87.1)	
**PVTT, *n* (%)**				
** I**	10 (14.5)	7 (18.4)	3 (9.7)	.670
** II**	23 (33.3)	11 (28.9)	12 (38.7)	
** III**	33 (47.8)	18 (47.4)	15 (48.4)	
** IV**	3 (4.3)	2 (5.3)	1 (3.2)	

Abbreviations: HBV, hepatic B virus; ECOG, Eastern Cooperative Oncology Group; AFP, alpha-fetoprotein; WBC, white blood cell; ALBI, Albumin-Bilirubin; PVTT, portal vein tumor thrombus; SBRT, stereotactic body radiotherapy; Sin + Bev, sintilimab + bevacizumab.

### Survival outcome and treatment response

**Figure 1.**
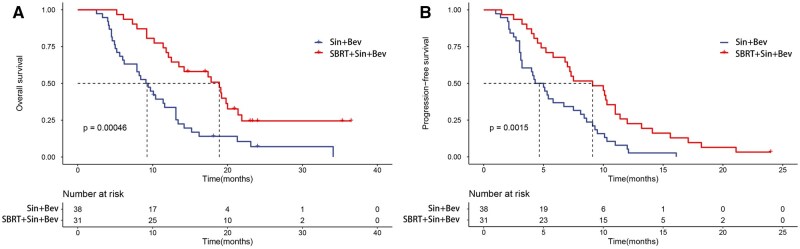
Kaplan–Meier curves for all patients treated with SBRT + Sin + Bev or Sin + Bev. (A) OS for overall patients. (B) PFS for overall patients. SBRT, stereotactic body radiotherapy; Sin + Bev, Sintilimab plus Bevacizumab; OS, overall survival; PFS, progression-free survival.

At the data cutoff date, the median follow-up time was 16.1 months in the SBRT + Sin + Bev group and 12.4 months in the Sin + Bev group, respectively. The SBRT + Sin + Bev group had a longer median OS and median PFS than the Sin + Bev group (median OS: 18.9 months vs 9.3 months, *P* < .001; median PFS: 9.1 months vs 4.7 months, *P* = .0015; Figure 1).

Treatment responses were presented in Table 2. The SBRT + Sin + Bev group demonstrated a significantly higher PR rate (58.1% vs 23.7%, *P* = .004) and a lower rate of progression disease (PD; 9.8% vs 31.6%, *P* = .027) than the Sin + Bev group. No significant differences were observed in CR (6.5% vs 2.6%, *P* = .857) or SD (38.7% vs 42.1%, *P* = .157) between the two groups. Consequently, the responses were significantly better in the SBRT + Sin + Bev group compared to the Sin + Bev group (ORR: 64.6% vs 26.3%, *P* = .031; DCR: 90.2% vs 68.4%, *P* = .028). Furthermore, patients in the whole cohort who responded to treatment were consistent with longer OS (*P* < .001; Figure S2).

**Table 2.** Best tumor response evaluated by mRECIST.

Characteristics	Total enrolled (*n* = 69)	Sin + Bev (*n* = 38)	SBRT + Sin + Bev (*n* = 31)	*P*
**Complete response, *n* (%)**	3 (4.3%)	1 (2.6%)	2 (6.5%)	.857
**Partial response, *n* (%)**	23 (33.3%)	9 (23.7%)	18 (58.1%)	.004
**Stable disease, *n* (%)**	31 (44.9%)	16 (42.1%)	8 (38.7%)	.157
**Progressive disease, *n* (%)**	15 (21.7%)	12 (31.6%)	3 (9.8%)	.028
**Objective response rate, *n* (%)**	26 (37.6%)	10 (26.3%)	16 (64.6%)	.031
**Disease control rate, *n* (%)**	57 (78.3%)	26 (68.4%)	28 (90.2%)	.028

Abbreviations: mRECIST, modified Response Evaluation Criteria in Solid Tumors; SBRT, stereotactic body radiotherapy; Sin + Bev, sintilimab + bevacizumab.

### Subgroup analysis

**Figure 2.**
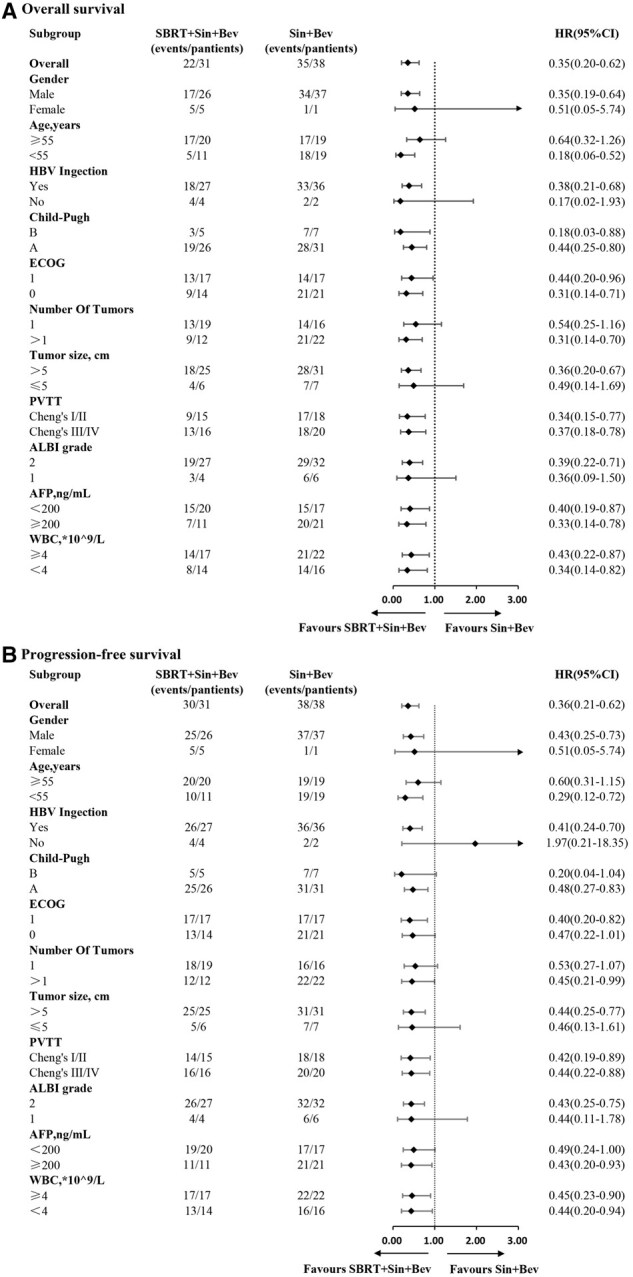
Forest plot of subgroup analyses for all patients treated with SBRT + Sin + Bev or Sin + Bev. (A) Subgroup analysis for OS. (B) Subgroup analysis for PFS. SBRT, stereotactic body radiotherapy; Sin + Bev, Sintilimab plus Bevacizumab; OS, overall survival; PFS, progression-free survival.

Forest plot analyses of OS demonstrated that patients treated with SBRT + Sin + Bev derived greater benefit than those treated with Sin + Bev alone across multiple subgroups. These included male sex, tumor size >5 cm, age <55 years, HBV infection, tumor number >1, ALBI grade 2, and all subgroups stratified by Child-Pugh, ECOG, PVTT classification, AFP level, and WBC count (Figure 2A). Similarly, PFS in the SBRT + Sin + Bev group were better in patients with the following characteristics: male sex, ECOG 1, Child-Pugh A, HBV infection, age <55 years, tumor number >1, tumor size >5 cm, ALBI grade 2, AFP ≥ 200 ng/mL, and across all PVTT and WBC subgroups (Figure 2B).

**Figure 3.**
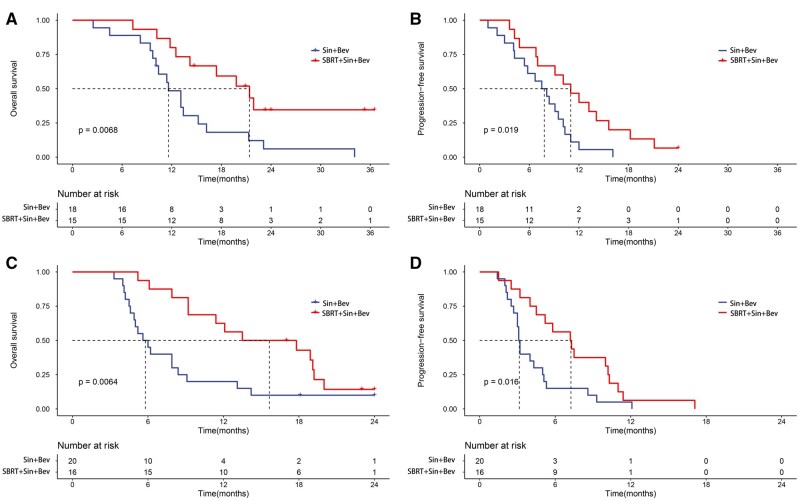
Kaplan–Meier curves of OS and PFS in patient subgroups stratified by PVTT classification. (A) OS in the PVTT type I/II subgroup. (B) PFS in the PVTT type I/II subgroup. (C) OS in the PVTT type III/IV subgroup. (D) PFS in the PVTT type III/IV subgroup. SBRT, stereotactic body radiotherapy; Sin + Bev, Sintilimab plus Bevacizumab; OS, overall survival; PFS, progression-free survival.

Whole patients were then classified into two PVTT subgroups (PVTT I/II and PVTT III/IV). In the PVTT I/II subgroup, the SBRT + Sin + Bev group demonstrated better OS and PFS than the Sin + Bev group. The median OS was 21.4 months (95% CI: 14.2–NA) vs 11.6 months (95% CI: 10.1–21.3; *P* = .0068; Figure 3A), and the median PFS was 11.0 months (95% CI: 7.0–18.2) vs 7.8 months (95% CI: 5.4–10.3; *P* = .019; Figure 3B). Similarly, in the PVTT III/IV subgroup, the SBRT + Sin + Bev group also showed improved outcomes, with a median OS of 15.7 months (95% CI: 9.2–NA) compared to 5.8 months (95% CI: 4.9–9.1; *P* = .0064; Figure 3C), and a median PFS of 7.3 months (95% CI: 4.5–11.0) vs 3.2 months (95% CI: 3.0–5.1; *P* = .016; Figure 3D).

### Prognostic factors

Univariate regression analysis identified SBRT + Sin + Bev and type I/II PVTT classification as significant risk factors improving both OS and PFS. Multivariate regression analysis identified SBRT + Sin + Bev treatment and type I/II PVTT classification significantly enhanced OS and PFS, with HR of 0.35 (95% CI: 0.20–0.62) for OS and 0.36 (95% CI: 0.21–0.62) for PFS. Additionally, Child-Pugh B significantly decreased PFS (Table 3).

**Table 3.** Univariate and multivariate Cox regression analysis on OS and PFS.

Variable	OS						PFS					
	Univariable			Multivariable			Univariable			Multivariable		
	HR	95%CI	*P*	HR	95%CI	*P*	HR	95%CI	*P*	HR	95%CI	*P*
**Gender (refer to female)**	0.72	0.31-1.70	.450				1.00	0.43-2.34	.993			
**Therapy (refer to Sin + Bev)**	0.39	0.23-0.67	.001	0.35	0.20-0.62	.000	0.45	0.27-0.74	.002	0.36	0.21-0.62	.000
**Age (refer to <55 years)**	1.10	0.65-1.87	.723				0.98	0.61-1.59	.934			
**Etiology (refer to HBV-negitive)**	0.98	0.42-2.30	.969				0.51	0.22-1.21	.127			
**Child–Pugh (refer to A)**	1.55	0.78-3.11	.212				1.82	0.97-3.43	.063	2.02	1.04-3.92	.038
**ECOG (refer to 0)**	0.95	0.56-1.61	.854				1.11	0.69-1.80	.669			
**Tumor number (refer to =1)**	1.59	0.94-2.70	.086	1.58	0.92-2.70	.097	1.51	0.93-2.46	.095	1.44	0.86-2.39	.164
**Tumor size (refer to <5 cm)**	1.18	0.60-2.30	.634				1.80	0.95-3.41	.071	1.38	0.67-2.81	.380
**PVTT (refer to Cheng’ I/II)**	1.83	1.07-3.11	.026	1.91	1.07-3.39	.028	2.16	1.31-3.55	.002	2.37	1.34-4.18	.003
**ALBI grade (refer to 1)**	0.89	0.43-1.81	.737				0.76	0.39-1.51	.435			
**AFP (refer to <200 ng/mL)**	1.03	0.61-1.73	.925				0.97	0.60-1.57	.898			
**WBC (refer to <4 × 109/L)**	1.68	0.98-2.87	.059	1.35	0.76-2.40	.307	1.54	0.95-2.49	.082	0.91	0.52-1.62	.757

Abbreviations: HBV, hepatic B virus; ECOG, Eastern Cooperative Oncology Group; PVTT, portal vein tumor thrombus; ALBI, Albumin-Bilirubin; AFP, alpha-fetoprotein; WBC, white blood cell; SBRT, stereotactic body radiotherapy; Sin + Bev, sintilimab + bevacizumab; OS, overall survival; PFS, progression-free survival.

### Safety

The overall incidence of TRAEs was comparable and manageable between the two groups (*P* = .605). There were eight cases (25.8%) in the SBRT + Sin + Bev group and six cases (15.8%) in the Sin + Bev group that experienced dose reduction or treatment discontinuation due to TRAEs (≥grade 3). Fatigue (14 cases [45.2%]) and elevated ALT/AST levels (9 cases [29.0%]) were the most common TRAEs in the SBRT + Sin + Bev group. In contrast, fatigue (15 cases [39.5%]) and hypertension (8 cases [21.1%]) were the most common TRAEs in the Sin + Bev group (Table 4). Three patients experienced grades 1-2 GI bleeding, with no ≥grade 3 events and no treatment discontinuations required.

**Table 4.** Treatment-related adverse events.

Adverse events	All grades		*P*	Grades ≥3		*P*
	Sin + Bev (*n* = 38)	SBRT + Sin + Bev (*n* = 31)		Sin + Bev (*n* = 38)	SBRT + Sin + Bev (*n* = 31)	
**Any adverse event, *n* (%)**	33 (86.8%)	29 (93.5%)	.605	6 (15.8%)	8 (25.8)	.303
**Fatigue, *n* (%)**	15 (39.5%)	14 (45.2%)	.634	0 (0%)	0 (0%)	-
**Rash, *n* (%)**	5 (13.2%)	4 (12.9%)	1.000	0 (0%)	0 (0%)	-
**Nausea/vomiting, *n* (%)**	5 (13.2%)	5 (16.1%)	.996	0 (0%)	0 (0%)	-
**Diarrhea, *n* (%)**	6 (15.8%)	5 (16.1%)	1.000	1 (2.6%)	2 (6.5%)	.857
**Hypertension, *n* (%)**	8 (21.1%)	8 (25.8)	.642	2 (5.3%)	3 (12.9%)	.813
**Proteinuria, *n* (%)**	2 (5.3%)	2 (6.5%)	1.000	0 (0%)	0 (0%)	-
**Constipation, *n* (%)**	2 (5.3%)	2 (6.5%)	1.000	0 (0%)	0 (0%)	-
**Hypothyroidism, *n* (%)**	4 (10.5%)	4 (12.9%)	1.000	0 (0%)	0 (0%)	-
**Immune-mediated hepatitis, *n* (%)**	0 (0%)	1 (3.2%)	.449	0 (0%)	0 (0%)	-
**GI haemorrhage, *n* (%)**	2 (5.3%)	1 (3.2%)	1.000	0 (0%)	0 (0%)	-
**Anorexia, *n* (%)**	6 (15.8%)	7 (22.6%)	.473	0 (0%)	1 (3.2%)	.449
**Anaemia, *n* (%)**	2 (5.3%)	5 (16.1%)	.277	1 (2.6%)	2 (6.5%)	.857
**Thrombocytopenia, *n* (%)**	4 (10.5%)	5 (16.1%)	.743	0 (0%)	1 (3.2%)	.449
**Leukopenia, *n* (%)**	2 (5.3%)	4 (12.9%)	.490	0 (0%)	1 (3.2%)	.449
**AST/ALT elevation, *n* (%)**	8 (21.1%)	9 (29.0%)	.444	2 (5.3%)	4 (12.9%)	.490
**Fever, *n* (%)**	3 (7.9%)	2 (6.5%)	1.000	1 (2.6%)	1 (3.2%)	1.000
**Hyperbilirubinemia, *n* (%)**	8 (21.1%)	7 (22.6%)	.878	2 (5.3%)	2 (6.5%)	1.000

Abbreviations: SBRT, stereotactic body radiotherapy; Sin + Bev, sintilimab + bevacizumab.

### Subsequent treatment

Subsequent treatments were generally comparable between the two groups. Specifically, tumor progression occurred in all 38 cases (100%) in the Sin + Bev group, and 22 cases (57.9%) received subsequent treatment, including 12 cases (36.4%) receiving single therapy and 10 cases (24.2%) receiving multiple therapies. Tumor progression occurred in 30 cases (96.8%) in the SBRT + Sin + Bev group, and 21 cases (67.7%) received subsequent treatment, including 9 cases (29.0%) receiving single therapy and 12 cases (38.7%) receiving multiple therapies (Table S1).

## Discussion

In this research, we preliminarily evaluated the outcomes of combining systemic therapy with SBRT in HCC with PVTT. We demonstrated that the addition of SBRT to Sin + Bev notably enhanced survival and ORR compared to Sin + Bev alone. Moreover, the combination therapy was safe and manageable. SBRT combined with Sin + Bev could be an option for those patients and warrants validation in large-scale, prospective trials.

Based on China’s guidelines of HCC with PVTT,^3^ systemic therapy and RT are recommended treatment options for HCC with PVTT. There is strong theoretical rationale supporting the combination of RT with systemic therapy. Previous studies have demonstrated that RT can remodel the tumor microenvironment by converting immunologically “cold” tumors into “hot” tumors, thereby enhancing the effectiveness of immune therapies.^18^^,^^19^ Additionally, anti-VEGF agents could normalize the vasculature, promoting immune cells’ penetration into tumors via enhanced blood flow.^20^^,^^21^ Conversely, vascular normalization may also alleviate tumor hypoxia, enriching oxygen levels within tumor cells, which can further enhance the radiosensitivity of tumors and promote a synergistic effect with RT.^18^ Moreover, HCC-associated PVTT is particularly sensitive to RT.^7^ Effective RT can degrade tumor thrombi, reducing the risk of continuous hematogenous dissemination of tumor cells. Restoration of portal vein blood flow following RT may also contribute to improved liver function, providing additional clinical benefit to patients.^22^

Currently, there are no studies comparing the efficacy of Sin + Bev alone versus SBRT combined with Sin + Bev for HCC. According to our research, the efficacy of Sin + Bev was comparable to that reported in the ORIENT-32 study^5^ (median OS: 9.3 months vs not reached; median PFS: 4.7 vs 4.6 months; ORR: 26.3% vs 25.0%). It is important to note, however, that the patient populations were not entirely comparable between the two studies. In our cohort, all patients had PVTT but no extrahepatic metastases, whereas in the ORIENT-32 study, 28% of patients had PVTT and 73% had extrahepatic metastases. These differences in disease burden may partly explain the similarity in observed survival outcomes despite differences in baseline characteristics. Recently, a single-arm study by Zhu et al. demonstrated that IMRT plus Sin + Bev achieved a median OS of 24.0 months and a PFS of 13.8 months, with an ORR of 58.7%. In contrast, the SBRT + Sin + Bev group in our study demonstrated slightly less favorable outcomes (median OS: 18.9 months; median PFS: 9.1 months; ORR: 64.4%). This may be attributed to the better-balanced baseline characteristics in the reference study compared to ours. For example, the reference study excluded patients with tumors larger than 10 cm or more than 3 lesions, whereas our study did not impose restrictions on tumor size or number. In addition, this difference may also be attributable to variations in radiotherapy techniques employed across studies. Specifically, in Zhu’s study, IMRT covered both intrahepatic tumors and PVTT lesions comprehensively, whereas in our study, SBRT targeted both intrahepatic tumors and PVTT in only five patients, with the majority receiving SBRT limited to PVTT alone. This difference in radiation field coverage may partially explain the disparity in outcomes, although further research is needed to validate our hypothesis. Another prospective study investigated the validity of SBRT plus camrelizumab and apatinib in treating HCC patients with PVTT.^23^ The median OS and PFS were 12.7 months and 4.6 months, respectively, with an ORR of 47.5%. Overall, the prognosis in that study appeared slightly poorer compared to SBRT + Sin + Bev. It is important to note that 53.3% (32/60) of patients in that study had extrahepatic metastases, whereas our study excluded patients with extrahepatic disease, which may partially explain the disparity in outcomes.

PVTT was an independent prognostic factor for HCC, with higher PVTT classifications correlating with poorer outcomes.^24^^,^^25^ In our study, higher PVTT classification was consistent with short OS and PFS. Based on PVTT classification, subgroup analyses revealed that both OS and PFS were significantly improved in the SBRT + Sin + Bev group compared to the Sin + Bev group across different PVTT subtypes. These findings suggest that the addition of SBRT can enhance the prognosis of HCC patients across varying classifications. However, the number of type I & IV (I: *n* = 8, IV: *n* = 3) patients was relatively small in our cohort, and therefore, these subgroup findings should be interpreted with caution and considered exploratory rather than conclusive. Furthermore, we observed that the SBRT + Sin + Bev regimen demonstrated consistent survival benefits across the vast majority of other clinical subgroups in our study. This highlights the broad applicability and potential utility of incorporating SBRT into treatment options for PVTT.

As to safety, the incidence of TRAEs was similar between the SBRT + Sin + Bev group and the Sin + Bev group. No unexpected complications were identified, and most TRAEs were manageable. These findings are consistent with Zhu’s study, which found that IMRT combined with Sin + Bev was well tolerated and safe for advanced HCC patients.^14^ In their study, the overall incidence of TRAEs was 95.7%, with severe TRAEs (≥grade 3) occurring in 65.2% of patients. Elevated ALT/AST levels were observed in more than 40.0% of cases. By comparison, in our study, the overall TRAEs rate was 93.5%, with 25.8% of patients experiencing grade ≥3 TRAEs, and elevated ALT/AST levels observed in 29.0% of cases. With broadly comparable baseline characteristics, both SBRT plus Sin + Bev and IMRT plus Sin + Bev appears to provide manageable safety profiles in this setting. Furthermore, another prospective study investigating the addition of SBRT to camrelizumab and apatinib for HCC reported an overall TRAEs rate of 100%, with severe TRAEs (≥grade 3) in 22.5% of patients.^23^ The incidence of elevated ALT/AST was 47.5%. It must be noted that, in that study, tumors were bigger, 53.3% of patients with extrahepatic metastases, and many patients had received prior treatments, all of which could have contributed to the higher incidence of liver function abnormalities compared to our study.

However, this study has several limitations. First, as a retrospective study, it is subject to selection bias and potential confounding. Although baseline characteristics were balanced and treatment decisions were made by a multidisciplinary tumor board (MDT), some patients who were clinically eligible for SBRT ultimately received Sin + Bev alone, likely due to nonclinical factors such as socioeconomic status, psychological concerns, or personal preferences. Second, extrahepatic metastases of the tumor were excluded in all patients in this study. As a result, the benefits of this triple therapy approach for patients with both PVTT and extrahepatic metastases remain uncertain. Third, this study was conducted in China, with 91.3% of patients in our cohort were HBV-positive. Thus, the generalizability of our findings to HCC patients with other underlying liver disease etiologies requires further confirmation in future studies. Fourth, the relatively small sample size reduced the statistical power to detect subtle differences, particularly in subgroup analyses, and may have increased the risk of type II errors. Given these limitations, the conclusions of our research require further validation in a multi-center prospective study with larger cohorts.

## Conclusions

In this retrospective study with a relatively small sample size, the combination of SBRT with sintilimab plus bevacizumab was associated with encouraging survival outcomes and manageable adverse events, suggesting a favorable safety profile. While these findings support the potential of this triple therapy, prospective randomized studies are needed to confirm the benefit of SBRT in this setting.

## Supplementary Material

oyaf439_Supplementary_Data

## Data Availability

The data underlying this article are available from the corresponding author upon reasonable request.
