# Supplementary material for: Real-world outcomes of stereotactic body radiotherapy plus sintilimab and bevacizumab for hepatocellular carcinoma with portal vein tumor thrombus
Source: Oncologist. 2026 Jan 6;31(2):oyaf439. doi: 10.1093/oncolo/oyaf439 (PMC12854084; doi:10.1093/oncolo/oyaf439)
Supplement: oyaf439_Supplementary_Data [file oyaf439_supplementary_data.zip › Supplementray Figure legends.docx]

**Supplementary Figure legends**

**Supplementary Figure S1.** Patient flowchart. HCC, hepatocellular carcinoma. PVTT, portal vein tumor thrombus. TACE, [Transcatheter arterial chemoembolization](https://www.bing.com/ck/a?!&&p=fb47df6e812b1e66JmltdHM9MTcxMTg0MzIwMCZpZ3VpZD0yYmY2Mjc3YS04OTUzLTZkNWMtMmQ4Zi0zNGZhODgxOTZjOTkmaW5zaWQ9NTI4OQ&ptn=3&ver=2&hsh=3&fclid=2bf6277a-8953-6d5c-2d8f-34fa88196c99&psq=tace&u=a1aHR0cHM6Ly9lbi53aWtpcGVkaWEub3JnL3dpa2kvVHJhbnNjYXRoZXRlcl9hcnRlcmlhbF9jaGVtb2VtYm9saXphdGlvbg&ntb=1). SBRT, stereotactic body radiotherapy; Sin+Bev, sintilimab+bevacizumab.

**Supplementary Figure S2**. Kaplan-Meier curves of OS in the entire cohort according to treatment response. CR, complete response; PR, partial response; SD, stable disease; PD, progressive disease; OS, overall survival.
