# Supplementary material for: Real-world outcomes of stereotactic body radiotherapy plus sintilimab and bevacizumab for hepatocellular carcinoma with portal vein tumor thrombus
Source: Oncologist. 2026 Jan 6;31(2):oyaf439. doi: 10.1093/oncolo/oyaf439 (PMC12854084; doi:10.1093/oncolo/oyaf439)
Supplement: oyaf439_Supplementary_Data [file oyaf439_supplementary_data.zip › Supplementary Figure S1.pdf]

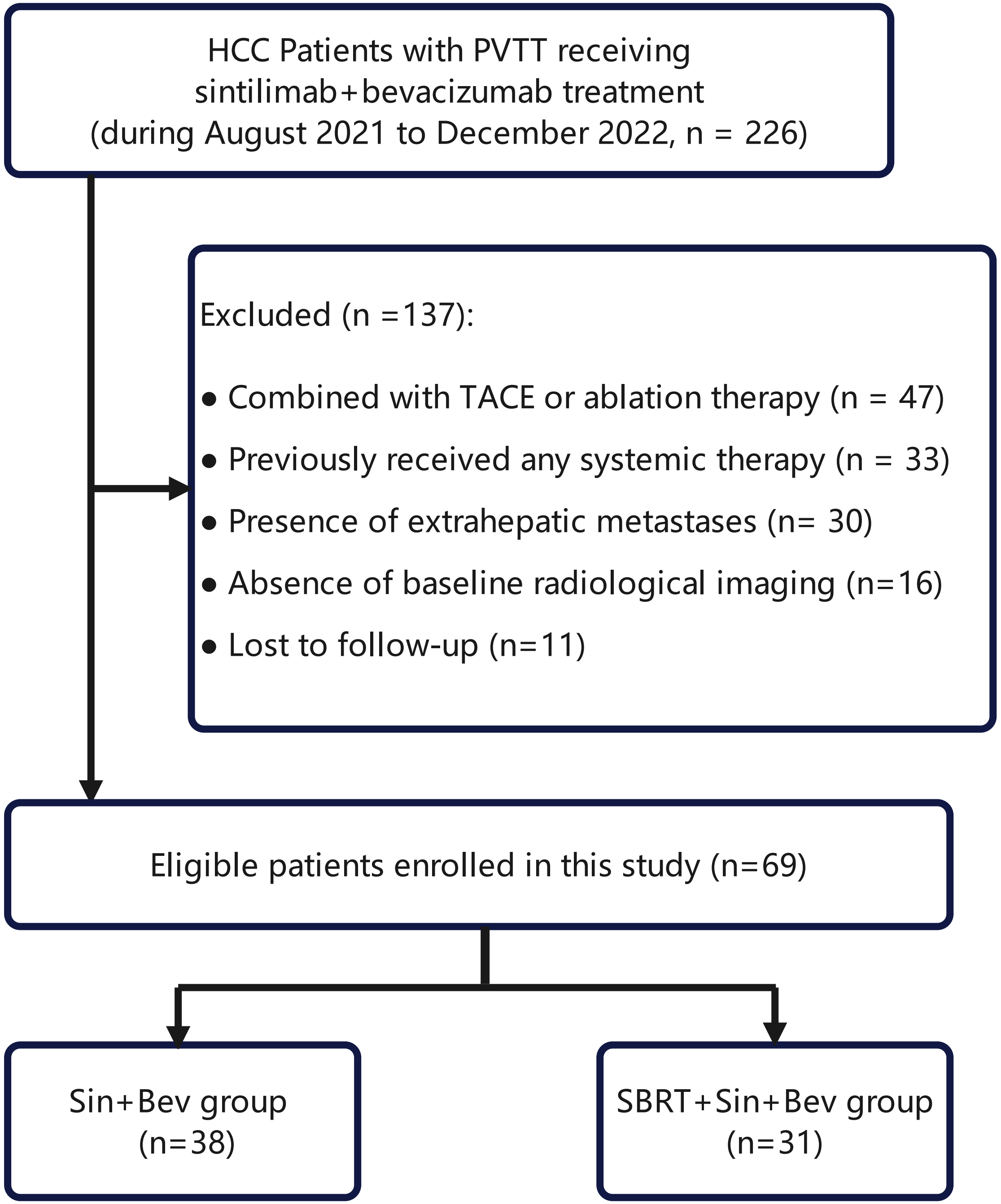
HCC Patients with PVTT receiving
sintilimab+bevacizumab treatment
(during August 2021 to December 2022, n = 226)
Excluded (n =137):
● Combined with TACE or ablation therapy (n = 47)
● Previously received any systemic therapy (n = 33)
● Presence of extrahepatic metastases (n= 30)
● Absence of baseline radiological imaging (n=16)
● Lost to follow-up (n=11)
Eligible patients enrolled in this study (n=69)
Sin+Bev group
(n=38)
SBRT+Sin+Bev group
(n=31)
