# Supplementary material for: Real-world outcomes of stereotactic body radiotherapy plus sintilimab and bevacizumab for hepatocellular carcinoma with portal vein tumor thrombus
Source: Oncologist. 2026 Jan 6;31(2):oyaf439. doi: 10.1093/oncolo/oyaf439 (PMC12854084; doi:10.1093/oncolo/oyaf439)
Supplement: oyaf439_Supplementary_Data [file oyaf439_supplementary_data.zip › Supplementary Table S1.docx]

**Supplementary Table S1.** Subsequent treatment

| **Treatment Programs** | **Sin+Bev**  **(n=38)** | **SBRT+Sin+Bev**  **(n=31)** | ***p*** |
| --- | --- | --- | --- |
|  |  |  |  |
| **Single treatment, n (%)** | 12 (31.6%) | 9 (29.0%) | 0.810 |
| TACE, n (%) | 3 (7.9%) | 6 (19.4%) | 0.299 |
| Regorafenib, n (%) | 3 (7.9%) | 0 (0%) | 0.247 |
| ICIs, n (%) | 6 (15.8%) | 3 (9.7%) | 0.500 |
| **Multiple treatments, n (%)** | 10 (26.3%) | 12 (38.7%) | 0.477 |
| TACE + ICIs, n (%) | 4 (10.5%) | 5 (16.1%) | 0.731 |
| Regorafenib + ICIs, n (%) | 2 (5.3%) | 1 (3.2%) | 1 |
| Lenvatinib + ICIs, n (%) | 1 (2.6%) | 3 (9.7%) | 0.336 |
| TACE + Lenvatinib + ICIs, n (%) | 3 (7.9%) | 3 (9.7%) | 1 |
| **Best Supportive Care, n (%)** | 16 (42.1%) | 10 (32.3%) | 0.379 |
| Abbreviations: TACE, Transarterial chemoembolization; SBRT, stereotactic body radiotherapy; ICIs, immune checkpoint inhibitors. | | | |
