# Supplementary figures and images for: Real-world outcomes of stereotactic body radiotherapy plus sintilimab and bevacizumab for hepatocellular carcinoma with portal vein tumor thrombus
Source: Oncologist. 2026 Jan 6;31(2):oyaf439. doi: 10.1093/oncolo/oyaf439 (PMC12854084; doi:10.1093/oncolo/oyaf439)

Overall survival

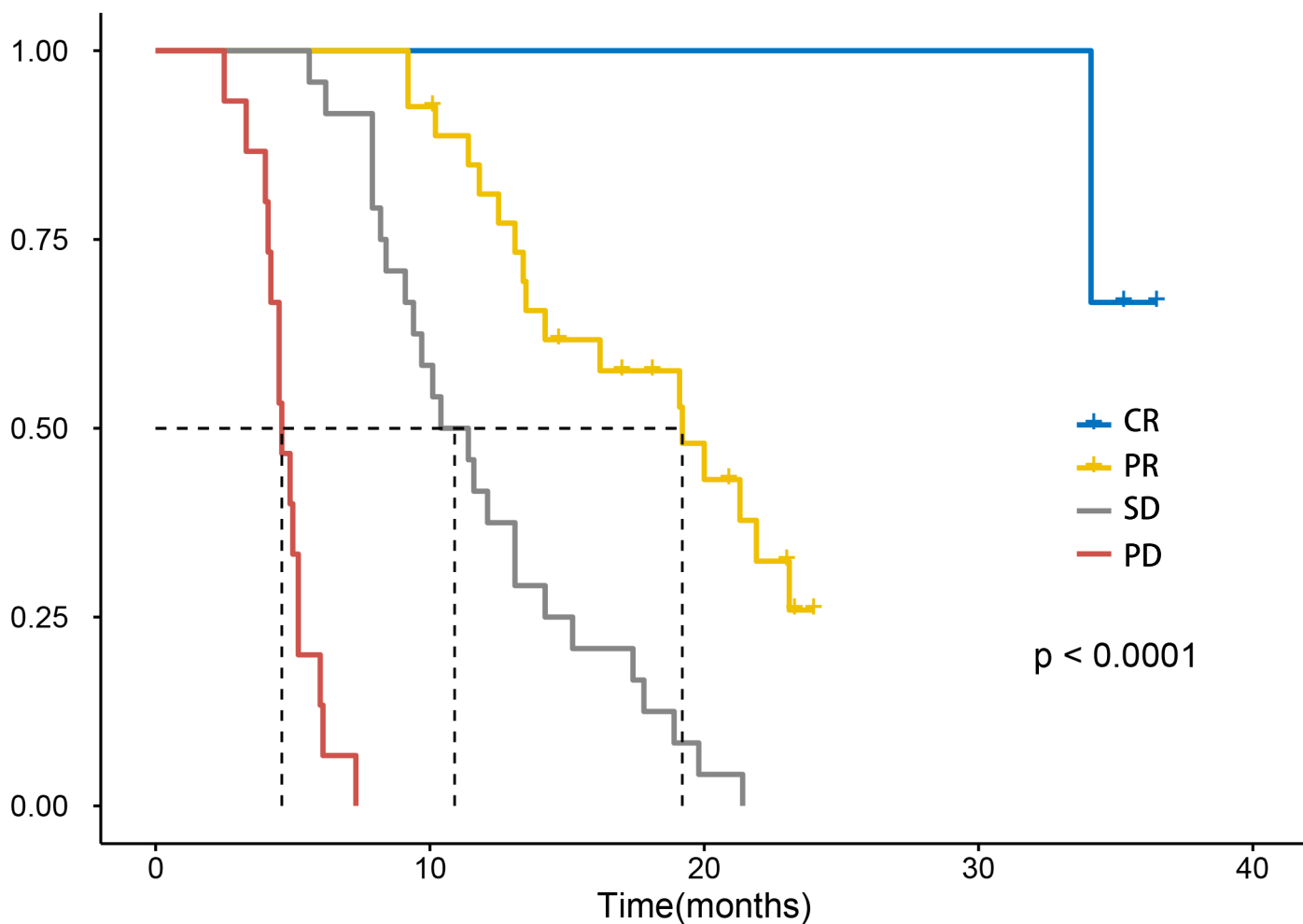

Number at risk

|    |              |    |    |    |    |
|----|--------------|----|----|----|----|
| CR | 3            | 3  | 3  | 3  | 0  |
| PR | 27           | 25 | 10 | 0  | 0  |
| SD | 24           | 14 | 1  | 0  | 0  |
| PD | 15           | 0  | 0  | 0  | 0  |
|    | 0            | 10 | 20 | 30 | 40 |
|    | Time(months) |    |    |    |    |

Supplement: oyaf439_Supplementary_Data [file oyaf439_supplementary_data.zip › Supplementary Figure S2.pdf]
